# Nodular Cystic Basal Cell Carcinoma of the Trunk: a Diagnostic Dilemma in an Unsuspecting Youth

**Published:** 2017-10-01

**Authors:** Ruchita Tyagi, Dilpreet Kaur, Gursheen Kaur, Bhavna Garg, Neena Sood, Sunil Gupta

**Affiliations:** 1 *Department of Pathology, Dayanand Medical College and Hospital, Ludhiana, India*; 2 *Department of Internal Medicine, Dayanand Medical College and Hospital, Ludhiana, India*; 3 *Department of Skin and venereal diseases, Dayanand Medical College and Hospital, Ludhiana, India*

**Keywords:** Basal Cell Carcinoma, Cystic, Nodular, Trunk

## Abstract

Basal cell carcinoma (BCC) commonly affects the elderly and is mostly confined to the head and neck region. Only 10% of all cases occur on the trunk. We presented a case of bullous lesion on the abdomen in a young male, initially diagnosed by clinicians as a vascular nevus. Histopathological examination and immunohistochemistry (IHC) revealed it to be Nodular cystic variant of BCC. This rare variant of BCC morphologically resembles benign skin adnexal tumor of Eccrine syringofibroadenoma. Ber Ep4 positivity on IHC established the correct diagnosis. This case highlights that nodular cystic variant of BCC can be a diagnostic dilemma for clinicians and pathologists.

## Introduction

Basal cell carcinoma (BCC) constitutes 70% of keratinocytic skin tumors.^1^ The incidence of BCC has been increasing worldwide. Although half of the cases of BCC occur in 50-80 years of age, the incidence in individuals less than forty years old has also been increased. BCC is rarely occurred in childhood and youth. ^2^ In 80-85% of the cases, BCC commonly develops in head and neck, the most common site are face, above *Onghren’s line *[line joining the angle of mouth and ear lobule]. Only ten percent of BCC are seen in trunk.^3^ However, in the recent years, an increase in the incidence of these lesions over the trunk has been noted.^2^ This case report highlights unusual presentation of BCC in a young individual over trunk, an uncommon location which was a diagnostic dilemma at presentation. 

## Case History

**Figure 1:**
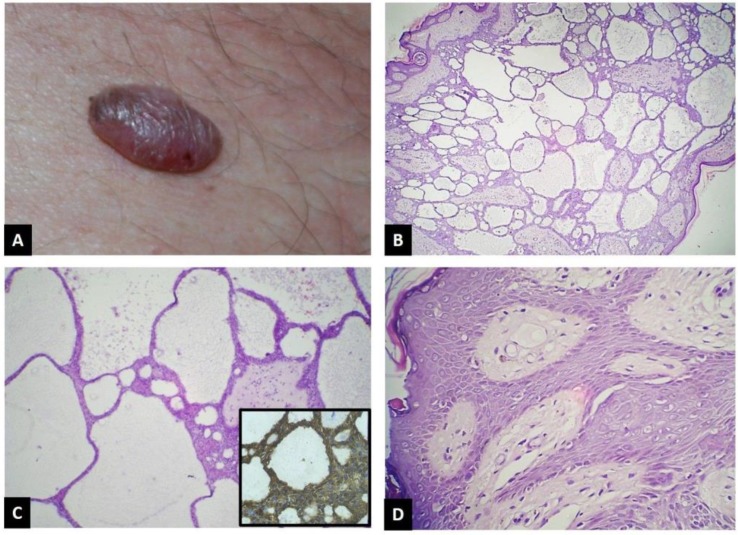
a - Clinical appearance of the lesion on trunk, b - Histopathology showing thin anastomosing epithelial cords of basaloid cells surrounded by fibro-myxoid stroma (H&E, 40x). c– Cysts, containing mucin at places, present between anastomosing cords of basaloid cells. (H&E, 100x) Inset showing Ber Ep 4 positivity of tumor cells on IHC, d- Peripheral pallisading also evident (black arrows) (H&E, 400x

A 27-year-old man presented with a bullous lesion over the trunk for the past five years, but now was slowly increasing in size. On examination, there was a well-defined erythematous bullous lesion measuring 2.5x1.5 cm on trunk, over the right lumbar region (Figure 1a). The clinical impression was a vascular nevus. Vitals, general examination and all routine investigations had normal findings on presentation. There were no such lesions elsewhere on the body. There was no significant family history. Histopathological examination (HPE) of excision biopsy showed presence of a tumor composed of thin anastomosing epithelial cords of atypical basaloid cells surrounded by fibromyxoid stroma (Figure 1b, c, d). The stroma between these anastomosing epithelial cords showed prominent cyst formation filled with mucin at places (Figure 1 b, c). Focally, peripheral pallisading was also evident (Figure 1d arrows). Morphologically, two possibilities were suggested as eccrine syringofibroadenoma [a rare benign skin appendageal tumor] and nodular cystic BCC. Immunohistochemistry (IHC) showed lesional cells with positive results for Ber- EP-4 and negative for EMA which ruled out the possibility of Eccrine syringofibroadenoma (Figure 1 c inset). Hence, the diagnosis of nodular cystic BCC was confirmed, which is an unusual variant of BCC on trunk. As the clinical impression of malignancy was not suspected initially, wide margin excision had not been performed and the tumor was infiltrating the resected margins and base of the specimen. Therefore, a wider excision was performed later, and the tissue submitted was free of tumor on HPE and showed mainly histiocytes and foreign body giant cell reaction, probably as a response to previous excision.

## Discussion

The occurrence of BCC in sun protected sites suggests association with many risk factors like skin type I, fair skin, red or blonde hair, blue or green eyes, freckling or sunburn in childhood, arsenic exposure, family history of skin cancer and immunosuppressive treatment. Predisposing genetic conditions include albinism, xeroderma pigmentosa, Bazex's syndrome and the naevoid basal cell carcinoma syndrome (Gorlin's syndrome). ^2^^,^^4^

Clinical subtypes of BCC include superficial, nodular, micronodular, infiltrating, fibroepithelioma. pigmented, morphea like and basosquamous.^1^^,^^2^^,^^4^ Nodular BCC is the most common, comprising 60-80% of the cases and occurs most commonly on head. It usually presents as elevated, exophytic pearl-shaped nodules with telangiectasia on the surface and periphery. Subsequently, it can extend into ulcerative or cystic pattern. Abdomen is an unusual location for nodular BCC. Clinicians can confuse BCC at first look, especially at an uncommon abdominal location, with malignant melanoma (pigmented basal cell carcinoma), melanocytic naevi (pigmented), sebaceous hyperplasia, molluscum contagiosum and appendageal tumours.^1^^,^^5^ In this case, BCC was never considered as a possibility on clinical examination.

Morphologically, nodular cystic variant of BCC can be confused with eccrine syringofibroadenoma which is a benign skin appendageal tumor composed of thin anastomosing epithelial cords of acrosyringeal cells, with or without lumen formation, embedded in a fibrovascular stroma. Ber Ep 4 positivity on IHC would establish the correct diagnosis. The skin appendageal tumor would have positive result for EMA and negative for Ber Ep 4.^1^^,^^5^^,^^6^

 Diagnosing such a lesion correctly, and in time, is of paramount importance because BCC can be locally aggressive. Besides, patients with basal cell carcinoma have an increased risk of developing basal cell carcinoma at other sites, and other skin cancers, such as malignant melanoma, squamous cell carcinoma and even non-cutaneous malignancies.^1^ In this case, the lesion was present for five years but the patient negligence in seeking medical attention resulted in delay of diagnosis. As there was no clinical suspicion of malignancy initially, wide margin excision was not performed the first time. The same was performed after confirmation of diagnosis on histopathology.

## Conclusion

This case highlights the occurrence of rare nodular cystic variant of BCC presenting in a young patient, an uncommon age, at an uncommon site; which can prove to be a diagnostic pitfall for clinicians as well as pathologists. The possibility of such an unusual diagnosis should be kept in mind while evaluating trunk lesions.
